# A new paracortinid millipede genus from caves in northern Vietnam, with descriptions of two new species (Diplopoda, Callipodida, Paracortinidae)

**DOI:** 10.3897/zookeys.1295.213545

**Published:** 2026-10-23

**Authors:** Hung-Anh Le, Dong V. Dang, Cuong T. Nguyen, Yen T. Phan, Nesrine Akkari, Anh D. Nguyen

**Affiliations:** 1 Institute of Biology, Vietnam Academy of Science and Technology, 18 Hoang Quoc Viet Road, Nghia Do, Hanoi, 10072, Vietnam Institute of Biology, Vietnam Academy of Science and Technology Hanoi Vietnam; 2 Hung Vuong University, Nguyen Tat Thanh Str., Nong Trang, Phu Tho, 29000, Vietnam Hung Vuong University Phu Tho Vietnam; 3 Zoological Department III, Natural History Museum Vienna, Burgring 7, 1010 Vienna, Austria Zoological Department III, Natural History Museum Vienna Vienna Austria

**Keywords:** Biodiversity, bio-inventory, cave fauna, karst region, Southeast Asia

## Abstract

A new genus, *Vietpetalum***gen. nov**., of the family Paracortinidae is here proposed to accommodate three paracortinid species from Vietnam: *V. marmoratum***sp. nov**., *V. simplex***sp. nov**., and *V. kyrang* (Nguyen, Stoev, Nguyen & Vu, 2023), **comb. nov**. A diagnosis of the genus is presented, and the new species are described. Cytochrome c oxidase subunit I (COI) barcodes are also provided for these three species.

## Introduction

The millipede family Paracortinidae Wang & Zhang, 1993 was initially described to accommodate the genus *Paracortina* Wang & Zhang, 1993 from southern China (Wang and Zhang 1993). Subsequently, several other taxa, discovered in China and Vietnam, have been described and assigned to this family (Zhang 1997; Shear 2000; Stoev 2004; Stoev and Geoffroy 2004; Liu and Tian 2015; Akkari et al. 2023; Chen et al. 2023; Nguyen et al. 2023). Recently, Akkari et al. (2023) further revised the taxonomy of the family, reviewed the secondary sexual characters and their potential importance in delimiting taxa, revalidated the genus *Scotopetalum* Shear, 2000, described the genus *Crassipetalum* Akkari & Stoev, 2023 and *C. magnum* Akkari & Stoev, 2023, and described two species *Paracortina asciformis* Akkari & Stoev, 2023 and *P. kabaki* Akkari & Stoev, 2023. Currently, the Southeast and East Asian family of Paracortinidae comprises 19 species classified in four genera, namely *Angulifemur* Zhang, 1997 (two species from caves in Yunnan, southern China), *Scotopetalum* Shear, 2022 (two species in northern Vietnam and southern China), *Crassipetalum* Akkari & Stoev, 2023 (two species in southern China), and *Paracortina* Wang & Zhang, 1993 (13 species in northern Vietnam and southern China). While redefining the taxonomy of the latter genus and the family based on the morphology of the gonopods, Akkari et al. (2023) expressed doubts on the generic position of two Vietnamese species, *Paracortina kyrang* Nguyen, Vu, Stoev & Nguyen, 2023 and *Paracortina multisegmentata* Stoev & Geoffroy, 2004. The recent discovery of two new species in Vietnam supports the hypothesis that some of the Vietnamese taxa form a morphologically distinct group that we here describe as a new genus, *Vietpetalum* gen. nov. The new genus, as we here propose, encloses three species: *Vietpetalum kyrang* (Nguyen, Vu, Stoev & Nguyen, 2023), comb. nov., *V. marmoratum* sp. nov., and *V. simplex* sp. nov. However, *P. multisegmentata*, which is also described from a cave in Vietnam, presents a different habitus and gonopod structure (e.g. proximally crossing telopodites) and its taxonomic position within Paracortinidae remains uncertain, as previously reported (Akkari et al. 2023).

## Material and methods

The millipede specimens were collected from caves in northern Vietnam and preserved in 90% ethanol. Morphological terminology follows Akkari et al. (2023). The holotype and most paratypes of the new species are deposited in the Myriapod collection of the Institute of Biology (IEBR-Myr), Hanoi, Vietnam.

Morphological characters were examined using an Olympus SZX10 stereomicroscope. Gonopods were dissected for a detailed morphological analysis and photographed. Colour images were taken at various focal planes using a Sony a6000 camera attached to a SMZ800N Nikon stereomicroscope. The images were stacked using Helicon Focus v. 7.0 and further processed in Adobe Photoshop CS6.

DNA was extracted using Qiagen DNeasy Blood and Tissue Kits. A 680-bp fragment of the mitochondrial gene, cytochrome c oxidase subunit I (COI), was amplified and sequenced using a pair of universal primers, LCO1490 and HCO2198 (Folmer et al. 1994). Polymerase chain reaction (PCR) conditions for amplification of the COI gene follow those of Nguyen et al. (2017). The successfully amplified PCR products were sent to the Phu Sa company (Vietnam) for purification and sequencing. COI sequences were verified using BLASTN v. 2.6.0+ (Zhang et al. 2000) and registered in GenBank with unique accession numbers.

### List of abbreviations:

**ap** = angular process on coxa 7;

**b** = subfalcate mesal process of coxa;

**Ca** = gonopodal coxal lobe;

**dl** = distal lamella;

**ep** = epiproct;

**hp** = hypoproct;

**Pf1, Pf2** = clavate prefemoroidal processes;

**pp** = paraproct;

**ps** = parasolenomere;

**PT** = pleurotergite;

**rp** = round process on coxa 7;

**sg** = seminal groove;

**sl** = solenomere;

**T** = telopodite.

## Results

### Order Callipodida

**Family Paracortinidae Wang & Zhang, 1993**

#### Genus Vietpetalum

Taxon classificationAnimaliaCallipodidaParacortinidae

Akkari & Nguyen gen. nov.

D2DE85C5-3535-5700-890A-A022CD88AA40

https://zoobank.org/BEEECC6C-D288-4CA6-897C-23ED65C5DF1C

##### Type species.

*Vietpetalum marmoratum* Le, Akkari & Nguyen, sp. nov.

##### Species included.

*Vietpetalum kyrang* (Nguyen, Stoev, Nguyen & Vu, 2023), comb. nov., *V. marmoratum* Le, Akkari & Nguyen, sp. nov., *V. simplex* Le, Akkari & Nguyen, sp. nov.

##### Diagnosis.

Head usually with a projection on vertex, leg-pair 6 without modifications; leg-pair 7 with a pair of a pointed mesal processes on coxa (seen in ventral view) and strong spines on trochanters. Gonopods mostly parallel, distally convergent, and pointing laterad. Each gonopod with 1 or 2 subequal prefemoroidal processes, a slender falcate process of coxa reaching distal part of telopodite; telopodite sinuous or curved, distally strongly narrowed, and bent *in situ* at an angle of 90° to main branch with apex downturned.

##### Differential diagnosis.

The new genus differs from all the other genera of Paracortinidae by having the telopodital stem strongly twisted and narrower, and its sinuous distal part mostly unbranched. The new genus is clearly different from *Angulifemur* by the absence of the characteristic angular projection on the telopodite. From *Crassipetalum* it differs by having much smaller prefemoroidal processes and much slenderer telopodite and subfalcate mesal coxal process. From *Scotopetalum* the new genus differs by the presence of the subfalcate mesal coxal process and from *Paracortina* by the much simpler shaped and narrowing distal part of telopodite, with a reduced to absent distal lamella.

##### Etymology.

The genus name derives from a combination of *Viet*, referencing Vietnam, and the suffix -*petalum*, which is commonly used in naming genera within the order Callipodida. Its gender is neuter.

##### Distribution.

The new genus is currently known to be endemic to Vietnam, occurring only in northern regions (Tuyen Quang and Cao Bang provinces).

#### Vietpetalum kyrang

Taxon classificationAnimaliaCallipodidaParacortinidae

(Nguyen, Stoev, Nguyen & Vu, 2023) comb. nov.

A539EEFB-2480-56D2-A32A-E442E4C167B2

Paracortina kyrang Nguyen, Stoev, Nguyen & Vu, 2023: 183, figs 1–7.

##### Differential diagnosis.

A species of the genus *Vietpetalum*, differing from congeners in having each gonopod with two clavate prefemoroidal process (Pf), the telopodite (T) sinusoidal, with two distal twists and strong constriction before the apex, and a distomesal spine (sp).

##### Remark.

Akkari et al. (2023) questioned the generic position of this species of *Paracortina* due to the derived configuration of the gonopods. This species is assigned here to *Vietpetalum***gen. nov**. together with two new congeners described below.

#### Vietpetalum marmoratum

Taxon classificationAnimaliaCallipodidaParacortinidae

Le, Akkari & Nguyen sp. nov.

4348195B-53F4-5CCB-9C54-0EF5EF9E175D

https://zoobank.org/C2B4A1E2-9B83-4CA2-BE86-B68839D70D51

Figs 1, 2, 3, 4, 5, 6, 7, 8, 16

##### Material examined.

***Holotype***: Vietnam – Tuyen Quang Province • male; Bach Xa commune, Cao Duong village, Bai Soi cave; 22.2878°N,104.9885°E; 630–700 m a.s.l.; 20 June 2026; Anh D. Nguyen, Yen T. Phan, Vu H. La leg.; IEBR-Myr 1081H.

**Figure 1.**
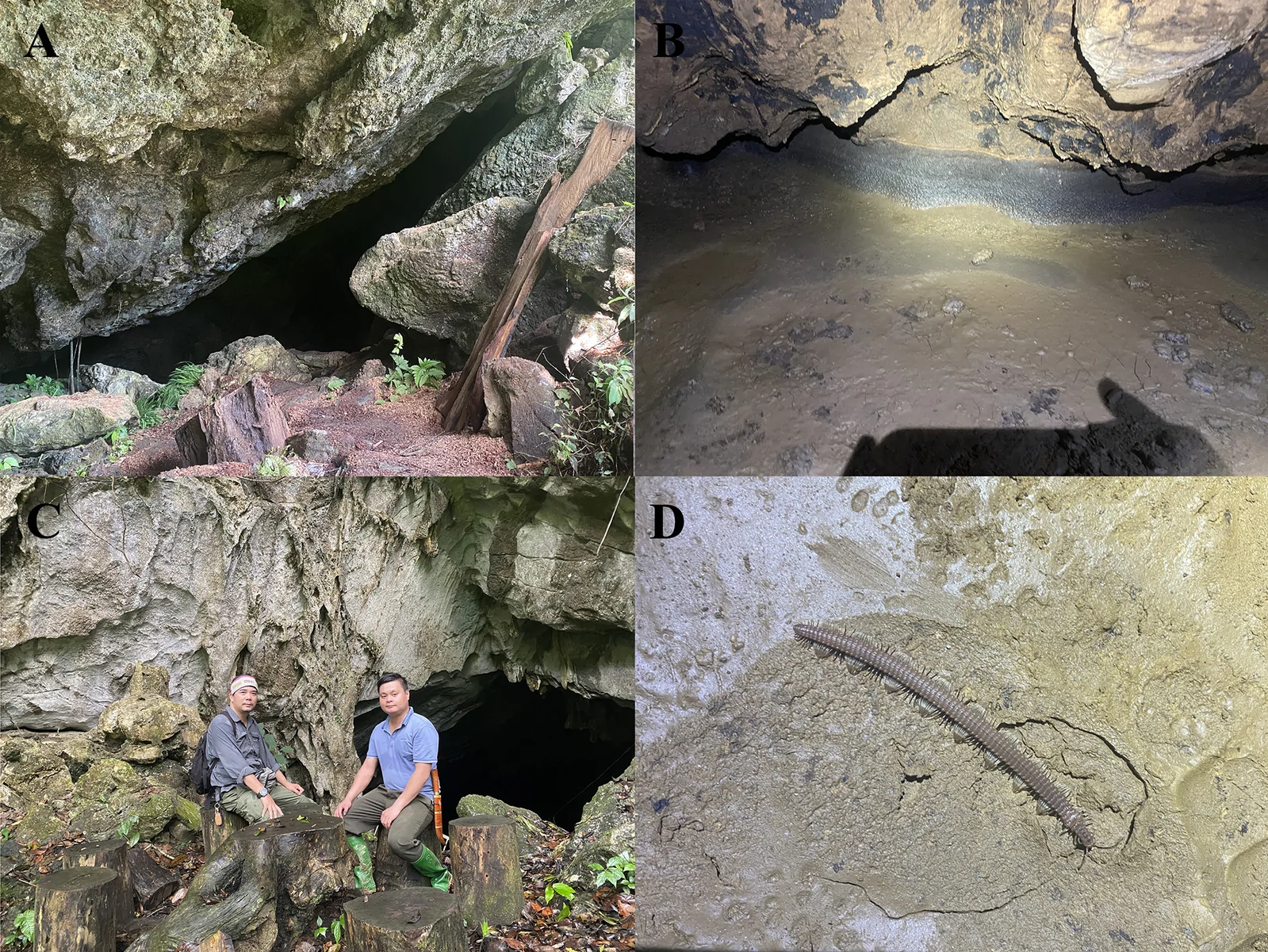
Habitats and habitus of *Vietpetalum marmoratum* sp. nov. **A**. Entrance of unnamed cave; **B**. Floor of unnamed cave; **C**. Bai Soi cave entrance with a forest ranger; **D**. Habitus of living specimen.

**Figure 2.**
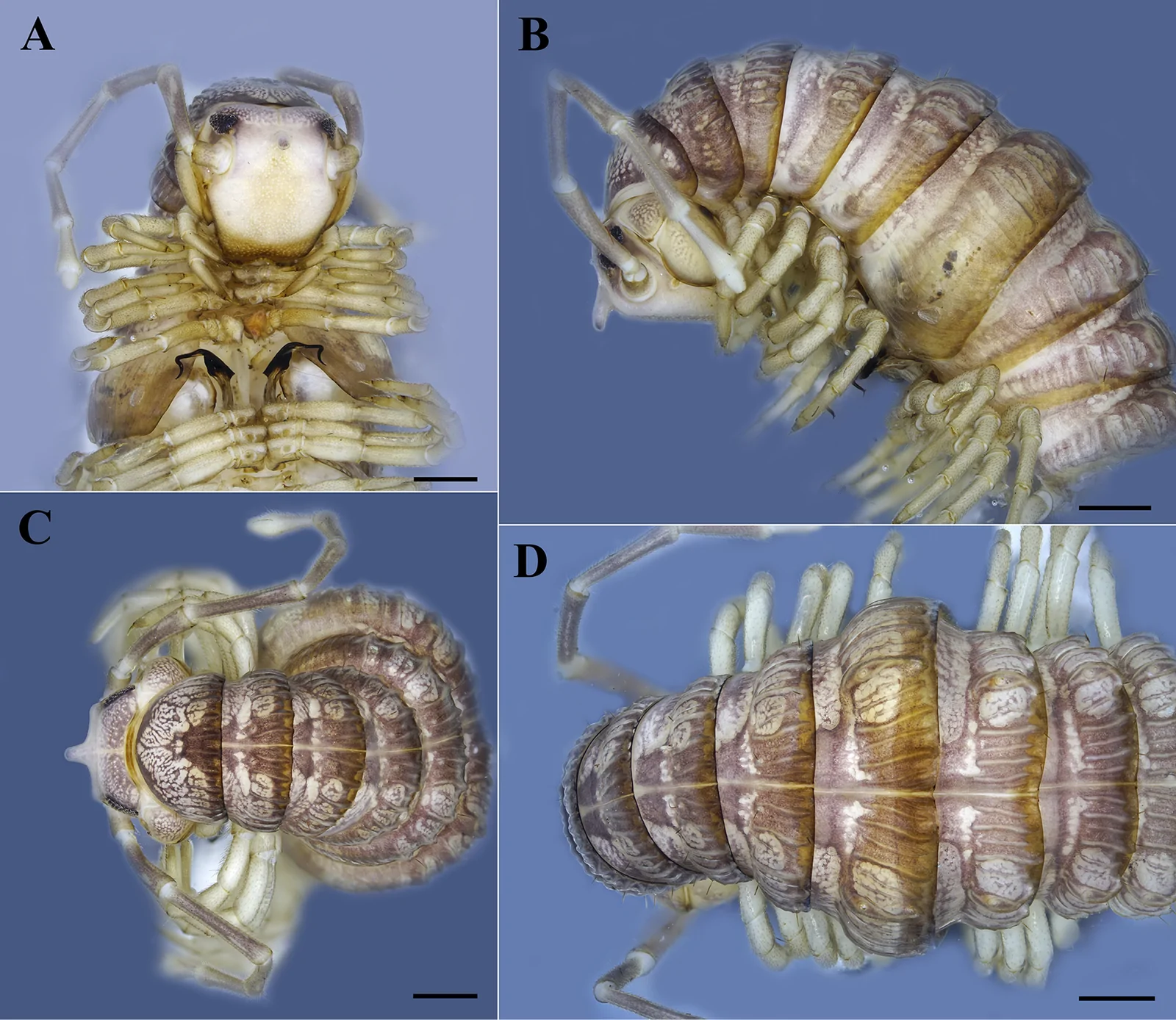
*Vietpetalum marmoratum* sp. nov., holotype, IEBR-Myr 1081H. **A–C**. Anteriormost part of body, ventral view (**A**), lateral view (**B**), dorsal view (**C**); **D**. Showing enlarged pleurotergites 3–7, dorsal view. Scale bars: 1 mm.

**Figure 3.**
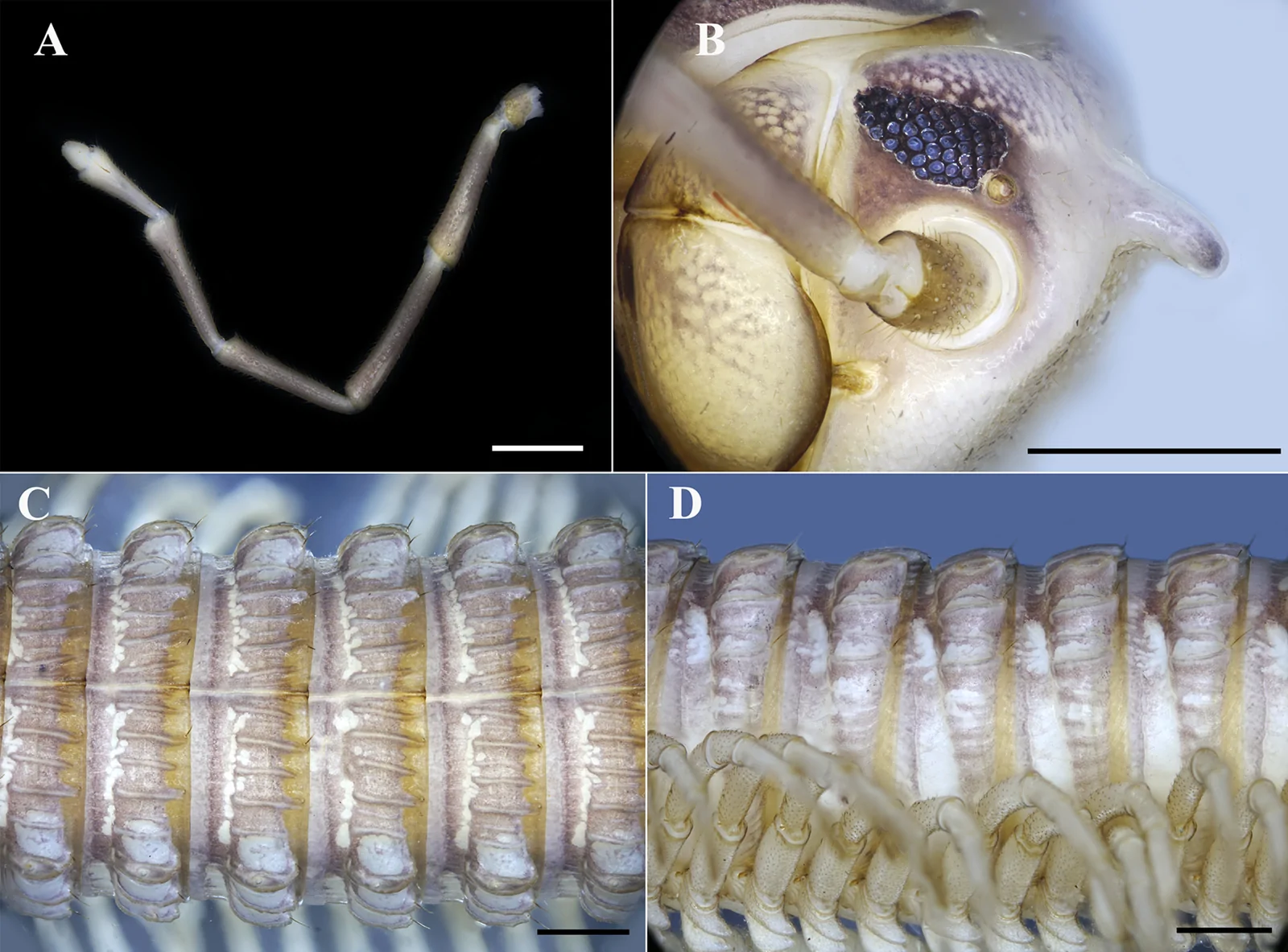
*Vietpetalum marmoratum* sp. nov., holotype, IEBR-Myr 1081H. **A**. Right antenna; **B**. Field of ommatidia; **C, D**. Midbody pleurotergites, dorsal view (**C**), lateral view (**D**). Scale bars: 1 mm.

**Figure 4.**
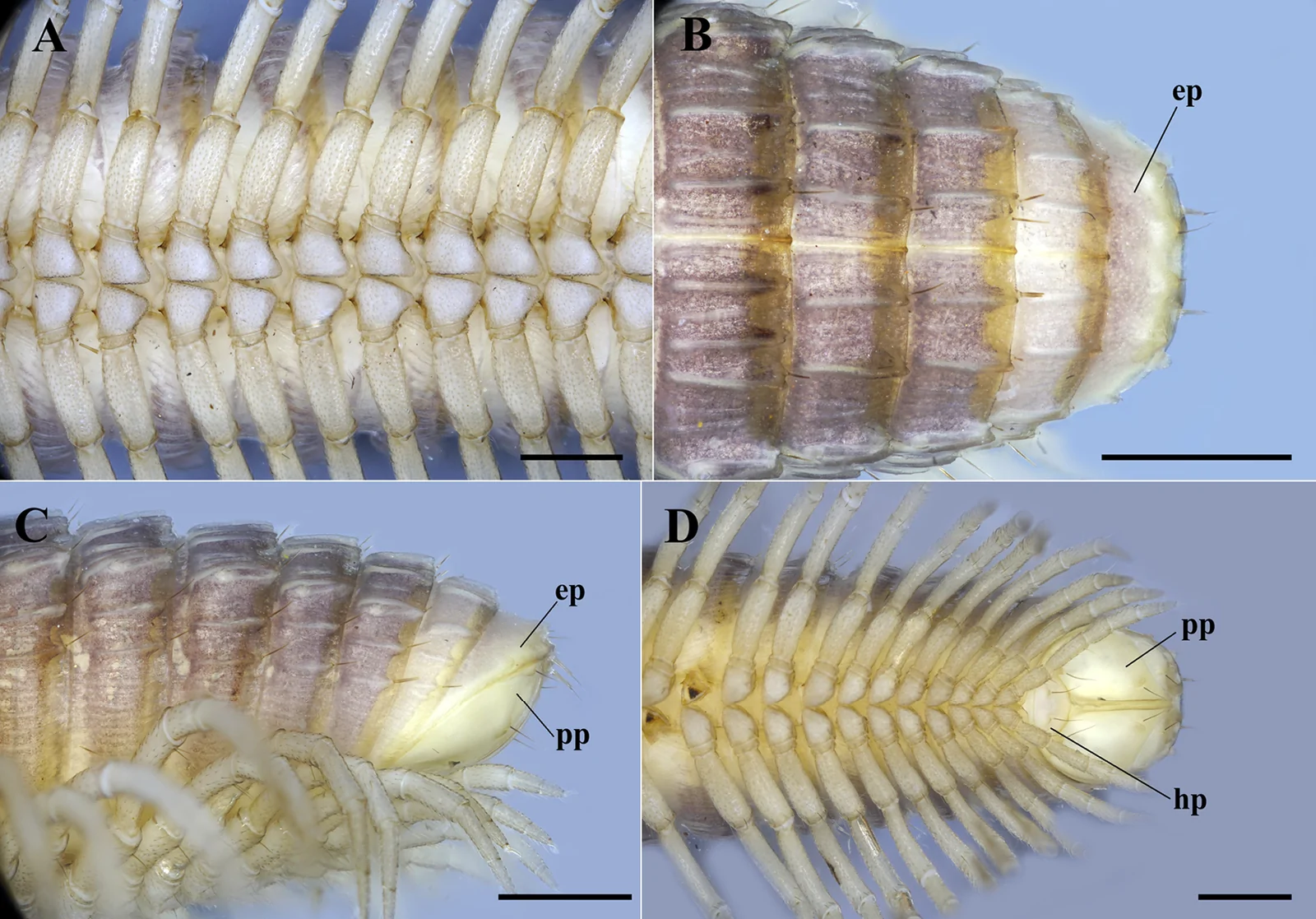
*Vietpetalum marmoratum* sp. nov., holotype, IEBR-Myr 1081H. **A**. Midbody pleurotergites, ventral view; **B–D**. Posteriormost pleurotergites and telson, dorsal view (**B**), lateral view (**C**), ventral view (**D**). Scale bars: 1 mm.

**Figure 5.**
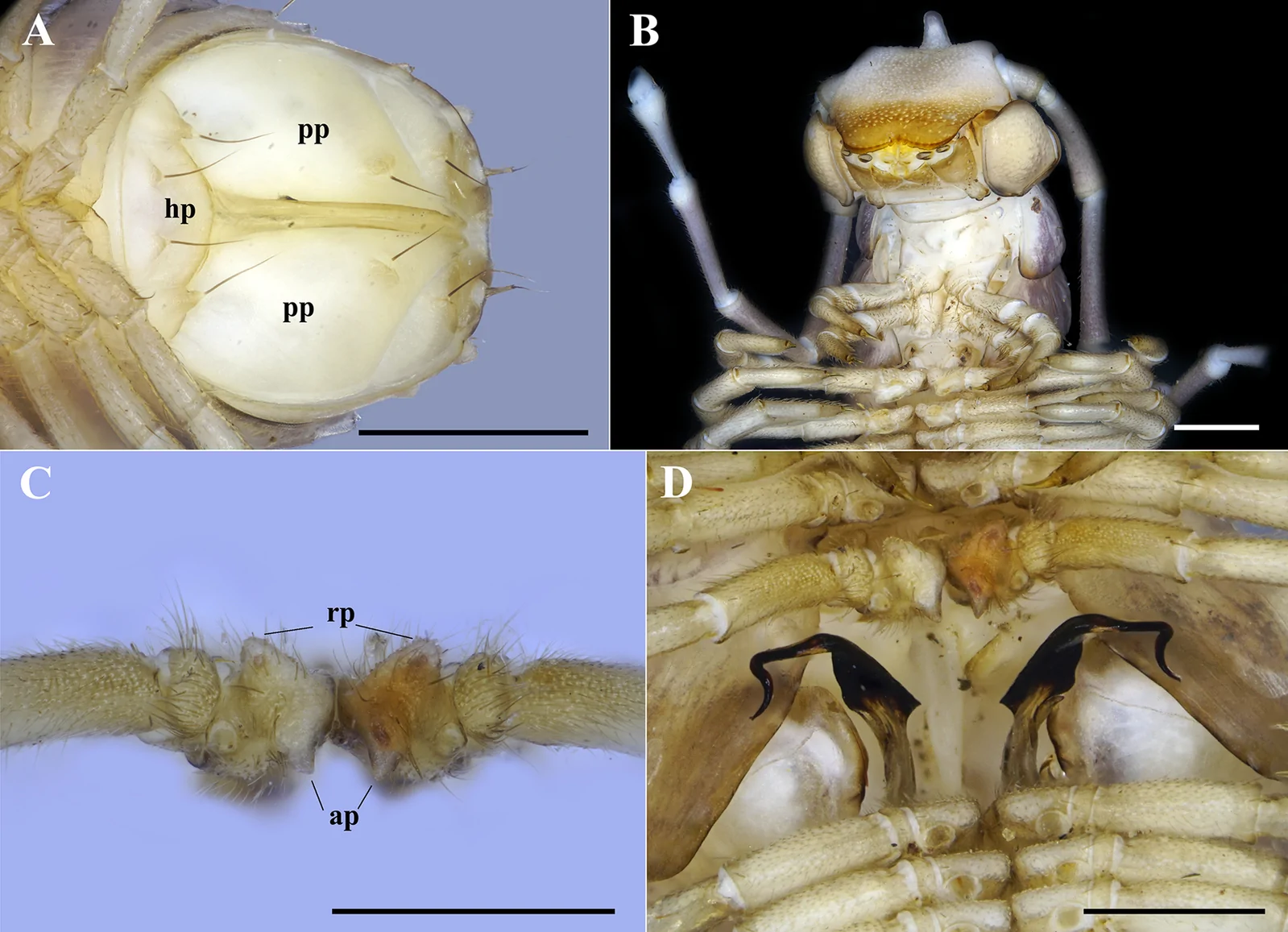
*Vietpetalum marmoratum* sp. nov., holotype, IEBR-Myr 1081H. **A**. Paraprocts and hypoproct; **B**. First and second pairs of legs; **C**. Legpair 7, coxa, trochanter, and part of the prefemur; **D**. Gonopod in gonocoel. Scale bars: 1 mm.

**Figure 6.**
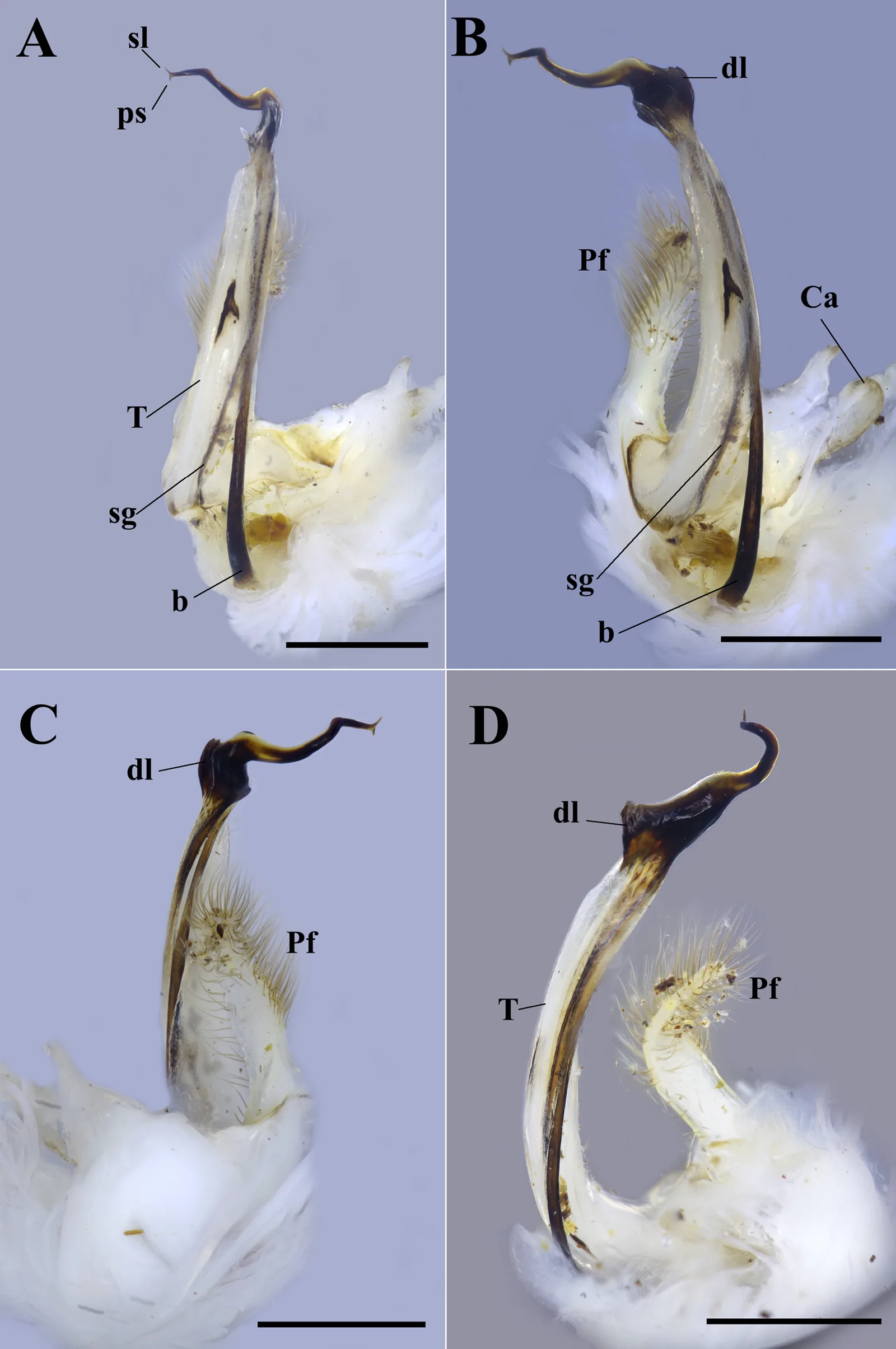
*Vietpetalum marmoratum* sp. nov. Holotype. IEBR-Myr 1081H. Right gonopod. **A**. Subventral view; **B**. Ventral view; **C**. Lateral view; **D**. Mesal view. Scale bars: 1 mm.

**Figure 7.**
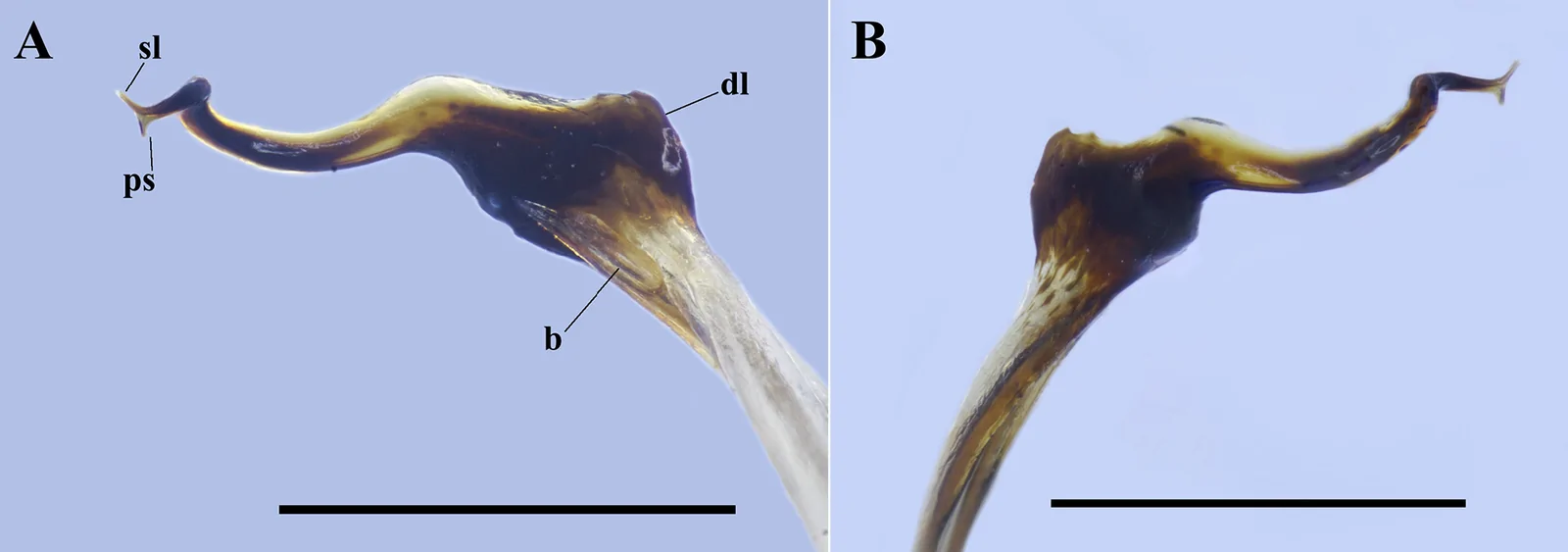
*Vietpetalum marmoratum* sp. nov., holotype, IEBR-Myr 1081H. **A, B**. Apical part of the gonopod, ventral view (**A**), lateral view (**B**). Scale bars: 1 mm.

**Figure 8.**
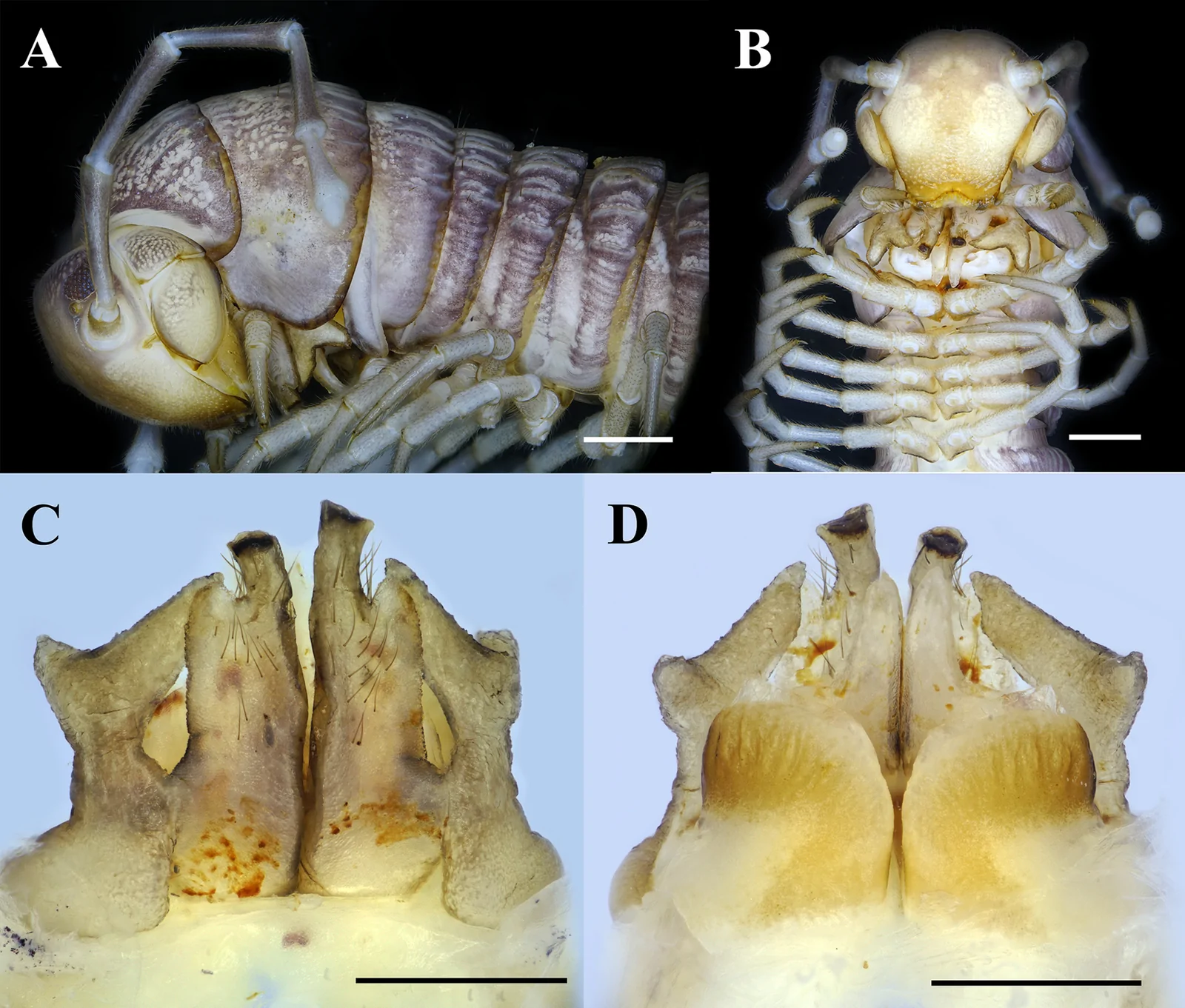
*Vietpetalum marmoratum* sp. nov., female, IEBR-Myr 1081H. **A, B**. Anteriormost part of body, lateral view (**A**), ventral view (**B**); **C, D**. Female sexual structures, anterior view (**C**), posterior view (**D**). Scale bars: 1 mm.

***Paratypes***: Vietnam • 1 female, 1 juvenile; same data as holotype; (IEBR-Myr 1081P).

***Non-type material***: Vietnam – Tuyen Quang Province • 1 male, 1 female, 1 subadult male; Bach Xa commune, Cao Duong village, cave near end of village; 22.3021°N, 104.9915°E, 630–700 m a.s.l.; 19 June 2026; Anh D. Nguyen, Yen T. Phan, Vu H. La leg.; IEBR-Myr 1080 • 2 females; ibid.; 17 December 2022; Anh D. Nguyen leg.; IEBR-Myr 1014 • 1 female, 3 juvenile; ibid. 21 April 2019; Anh D. Nguyen leg.; IEBR-Myr 799.

##### Differential diagnosis.

This species of *Vietpetalum* differs from congeners in having each gonopod with two clavate prefemoroidal processes (Pf) and the telopodite (T) distally curved at a 90° angle, with the apex downturned and with no distomesal spine.

##### Etymology.

The epithet marmoratum is from a Latin adjective, meaning “marbled”, used to emphasize the marbled pattern on collum and tergites 2–4.

##### Description.

**Male holotype**: length about 70.6 mm, width and height at midbody PT 2.7–2.8 mm and 2.9–3.0 mm, respectively; 72 PT+ telson.

**Male paratype**: 73PT + telson.

***Colour***: living specimens brownish grey (Fig. 1D). Ethanol-preserved specimens (Figs 2, 3, 4) generally reddish brown, with whitish-yellow marbled spots near primary crest 3; posterior part of metazonites with a brownish-yellow margin; head, pleurotergites, antennae, and telson yellowish brown; legs brownish yellow; body crests paler.

***Head*** (Fig. 2A–C) modified; frons with a strong projection; forehead and vertex concave.

***Antennae*** (Figs 2A–C, 3A) long, extending beyond posterior edge of PT 7 when folded backwards; ratio antenna/body length about 1/7; all antennomeres white; length of antennomeres: 1 = 0.5 mm; 2 = 1.5 mm; 3 = 1.9 mm; 4 = 1.6 mm; 5 = 1.8 mm; 6 = 1.3 mm; 7 = 0.5 mm; tip of antennomere 7 with four cones protruding well beyond the edge (not visible in Fig. 3A).

***Field of ommatidia*** (Fig. 3B) black, well delineated, composed of 48 ommatidia in eight vertical rows. Tömösváry’s organ about two times larger than the largest ommatidium, placed between eye and base of antenna (Fig. 3B).

Width of PT: 6>>7>8–14>5>4=3=2>1. PT slightly higher than broad; height of 10^th^PT: 3.00 mm, width 2.8 mm.

***Collum*** (Fig. 2B, C) much narrower than head; pleurotergite 6 in males strongly enlarged (Fig. 2A–D). Crests on collum (Fig. 2C) moderately expressed mostly in posterior part of ring. Complete crests series appearing from PT2 onwards. Above ozopores, midbody PT with 3+3 primary crests and with 3–5+3–5 secondary slightly shorter crests between primary crests (Figs 2C, 2D, 3C, 3D, 4B, 4C); 3^rd^ primary crest strongly enlarged, other primary crests not flattened, almost equally broad along metazonal length; secondary crests slightly shorter, but strong as primary crests. Ozopores lying on primary crest 3, visible from sixth to last but one PT (Figs 2C, 2D, 3C, 3D, 4B, 4C).

Below ozopores, midbody PT with 2+2 primary crests and 2+2 shorter and thinner secondary crests between primary crests, and 9–10 lower crests down to ventral pleurotergal edge (Figs 2B, 3D, 4C).

Midbody pleurotergal setae 5+5, located at caudal edges of primary crests (Figs 2, 3, 4); setal pattern as in Table 1. Axial line obviously distinct.

**Table 1.** Chaetotaxy in *Vietpetalum marmoratum* sp. nov.

	**Anterior setae**	**Posterior setae**
Collum	3?+3?	—
PT2	5+5	—
PT3	5+5	—
PT4	5+5	—
PT5	2+2	3+3
PT6–penultimate PT		5+5

***Epiproct*** (ep) (Fig. 4B, C) simple, with 2+2 setae on lateral sides and 2+2 setae in transverse rows on dorsum. Hypoproct (hp) (Figs 4D, 5A) tripartite, medial sclerite largest, subtrapeziform, bearing two paramedian macroseta; each lateral sclerite with a single macroseta born on a large tubercle. Paraprocts (pp) (= anal valves) (Figs 4C, 4D, 5A). smooth, each divided into a small upper and a large lower sclerite, both with a pair of macrosetae. Spinnerets long and slender, tube-shaped, each ending with a long seta. All setae on telson brown, contrasting with yellowish background.

***Male leg-pairs*** 1 and 2 much shorter, with strong setae on ventral side of femorite and tibia; leg-pair 3 slightly shorter than following legs; tarsal pads large visible on pregonopodal legs, then gradually thinner and eventually absent on subsequent legs. All legs ending with a rather slender, long, curved claw. Coxal sacs present from legs 3–23.

Coxa 2 with a pair of gonopores placed on small cones (Fig. 5B). Coxa 6 normal, without processes or modifications. Coxa 7 (Fig. 5C, D) with anterior round tuberculiform process (rp) directed ventrad and an angular process (ap) directed posteriad. Coxae of the remaining legs normal.

***Gonopods*** (Figs 6, 7) brownish yellow, some parts (seminal groove, distal part of telopodite) dark brown to black. Gonopods protruding well beyond the gonocoel, stems of telopodites long, thin, subparallel, and diverging, pointing laterad (Figs 5D, 6A–D). Each gonopod with a long, subfalcate prefemoroidal process (pf) densely apically covered with long macrosetae (Fig. 6B–D). Coxa with a round lobe (Ca) and a long, falcate spiniform process (b) (Fig. 6A, B), reaching the distal part of the telopodite. Telopodite (T) long, slender, apically twisted laterad (Figs 6B–D, 7A, 7B). Distal part of telopodite rather sinuous with a reduced lamella (dl), smoothly narrowing towards apex, bifurcating into solenomere (sl) and parasolenomere (ps) (Figs 6, 7). Seminal groove (sg) terminating in solenomere (Fig. 7).

**Female**: head unmodified (Fig. 8A, B). Length about 73.2 mm. 10^th^PT ca 2.9 mm wide and 3.1 mm high; 73–78 PT + telson. Leg-pair 2 strongly modified (Fig. 8B–D); coxa well developed, large, trapeziform, with a small, round tubercle, a highly elevated, chimney-shaped process, and anteriorly large spine-like process (Fig. 8C); other podomeres strongly modified as large, bilobed processes (Fig. 8C). Leg-pairs 1–3 with tarsal brushes. Cyphopods large and round (Fig. 8D). Coxae 7 normal, without processes.

##### DNA barcode.

The COI barcode is uploaded to GenBank with the accession number PQ685116. The new species has a genetic similarity of 82.86% with *Abacion magnum* (Loomis, 1943) (JN656584).

##### Cave habitat.

Millipede specimens were collected from two somewhat horizontal caves located approximately 1.5 km apart. Both caves are located at an elevation of 600–700 m a.s.l. Both caves have small, narrow entrances that widen inside. Darkness begins about 10 m from the entrance. The cave floor is mostly wet and covered with small rocks. Clay deposits were observed in the area where the species was discovered. The new species was found on clay ground and large rocks 100–200 m from the cave entrances. Callipodidans, glyphiulid millipedes, and ground beetles were also present in the caves alongside the new species.

##### Distribution.

The species is known only from its type locality.

##### Remarks.

We collected specimens of this species on three occasions. During the first survey (April 2019), only subadult males were found. In the second survey (December 2022), only females were collected. In the third survey (June 2026), both mature males and females were present. These observations suggest that the maturation period is likely to extend from June to October or November each year.

#### Paracortina simplex

Taxon classificationAnimaliaCallipodidaParacortinidae

Le, Akkari & Nguyen sp. nov.

4C5E51E2-68DC-54DF-B37B-BA7C00CBACAB

https://zoobank.org/43EFAFF1-B691-4441-AE48-E4C0E7179B1E

Figs 9, 10, 11, 12, 13, 14, 15, 16

##### Material examined.

***Holotype***: Vietnam – Tuyen Quang Province • male; Tùng Vài commune, unnamed cave; 23.08860°N, 104.80761°E; 1800 m a.s.l.; 19 May 2026; Anh D. Nguyen, HA Le, DV Dang, CT Nguyen leg.; IEBR-Myr 1070H.

**Figure 9.**
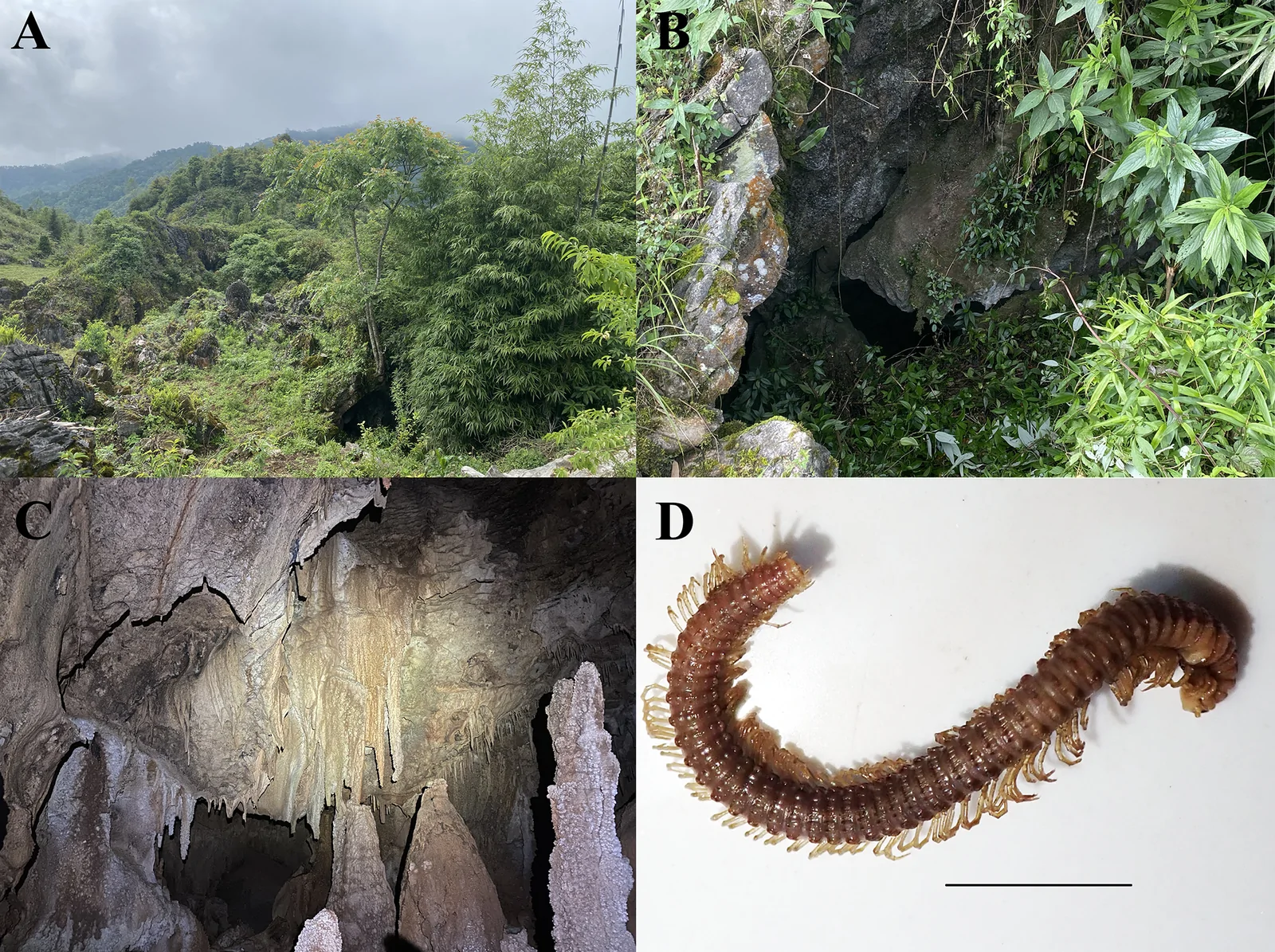
Habitats and habitus of *Vietpetalum simplex* sp. nov. **A, B**. Entrance of unnamed cave; **C**. Cave tunnel; **D**. Photograph of a preserved specimen. Scale bar: 5 mm.

**Figure 10.**
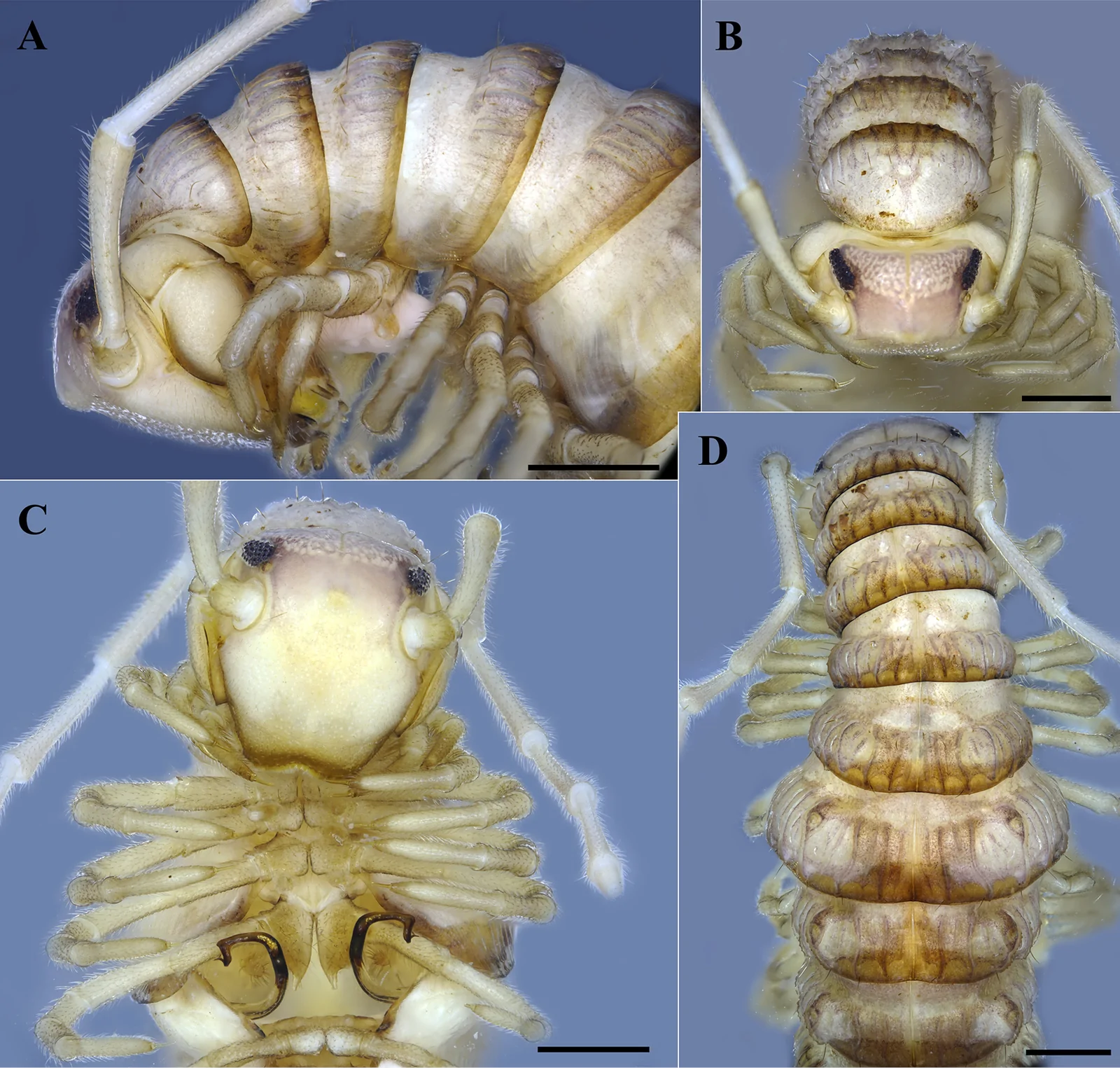
*Vietpetalum simplex* sp. nov., holotype, IEBR-Myr 1070H. **A–C**. Anteriormost part of body, lateral view (**A**), dorsal view (**B**), ventral view (**C**); **D**. Showing enlarged pleurotergites 2–7, dorsal view. Scale bar: 1 mm.

**Figure 11.**
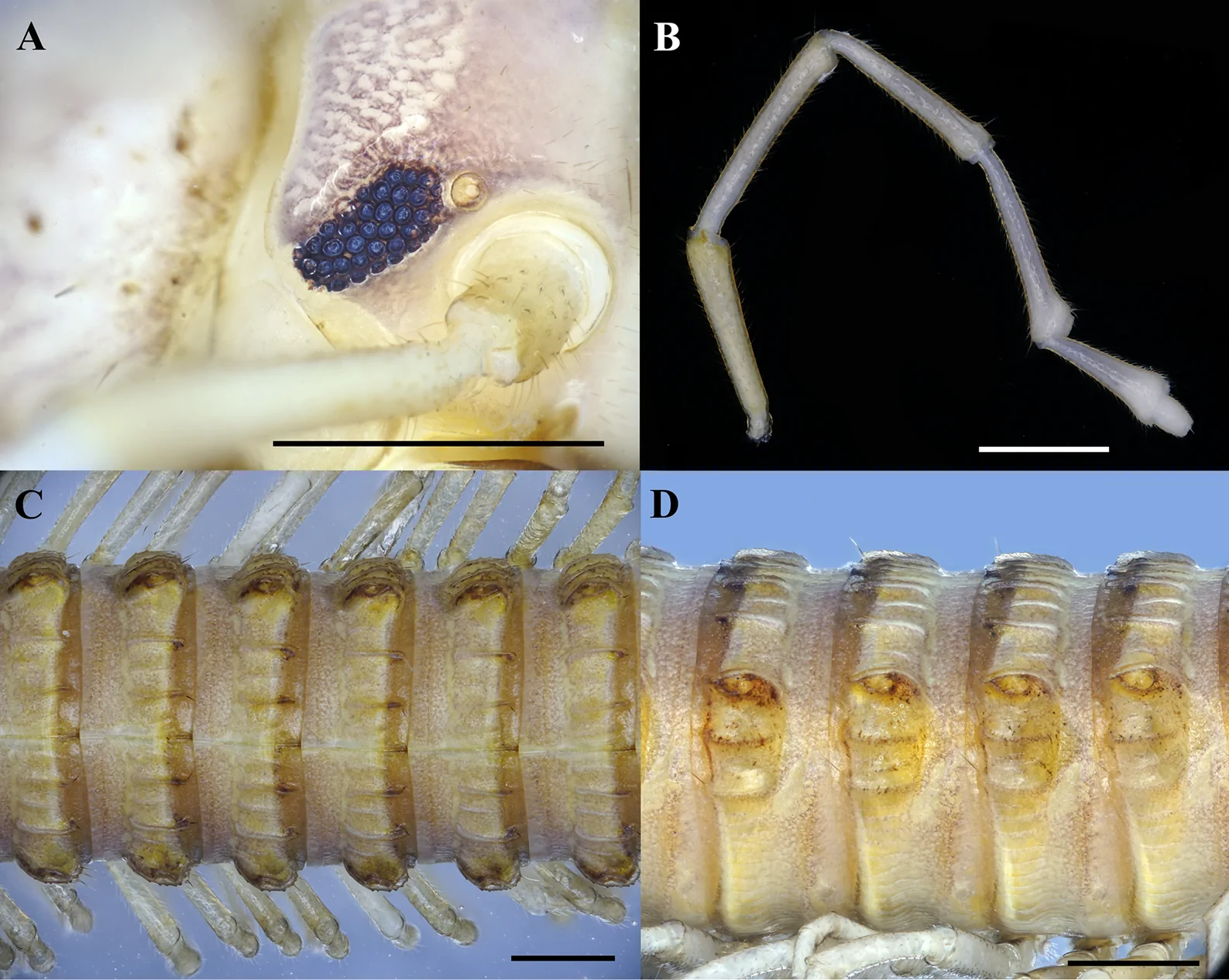
*Vietpetalum simplex* sp. nov., holotype, IEBR-Myr 1070H. **A**. Field of ommatidia; **B**. Right antenna; **C, D**. Midbody pleurotergites, dorsal view (**C**), lateral view (**D**). Scale bar: 1 mm.

**Figure 12.**
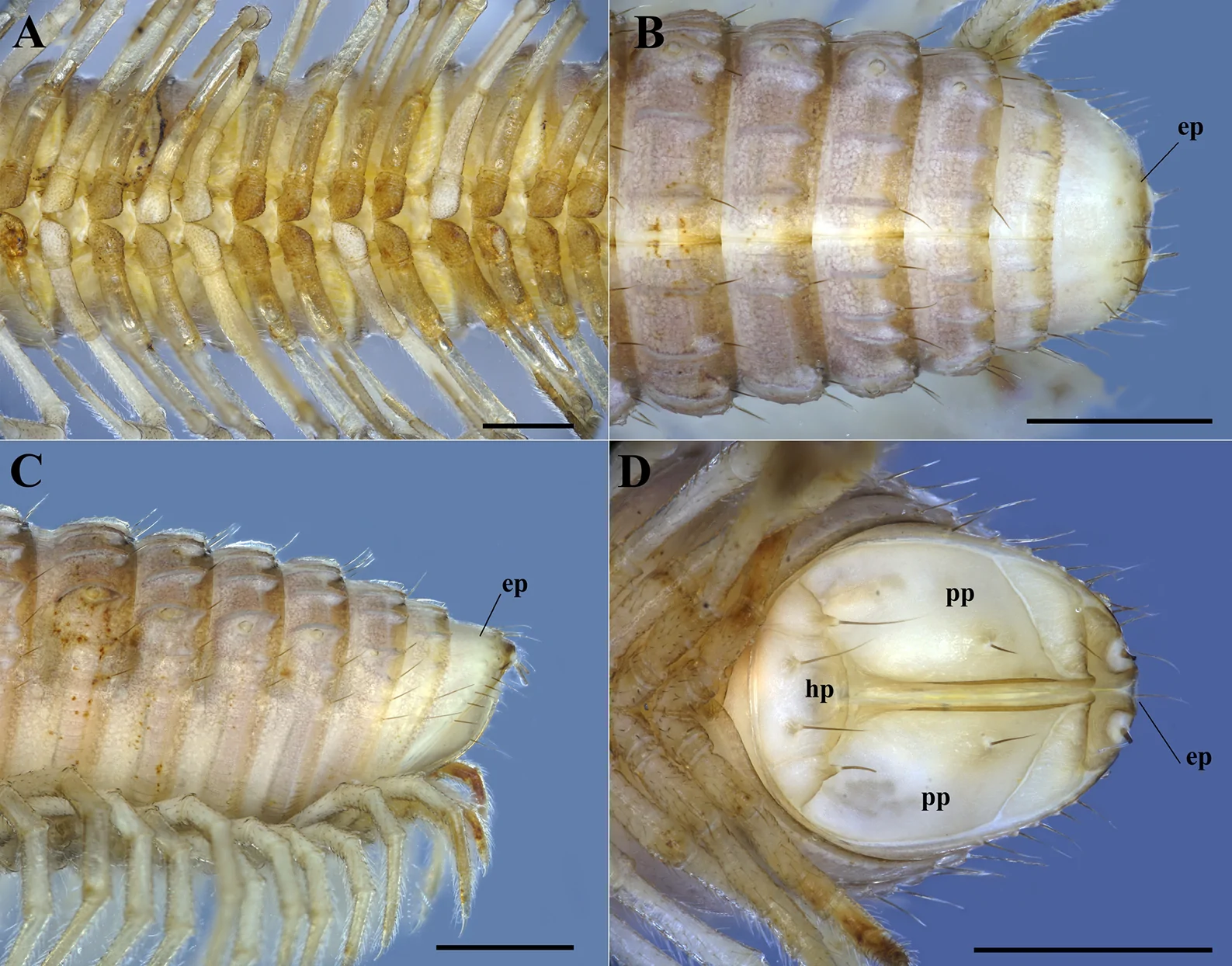
*Vietpetalum simplex* sp. nov., holotype, IEBR-Myr 1070H. **A**. Midbody pleurotergites, ventral view; **B, C**. Posteriormost part of body, dorsal view (**B**), lateral view (**C**); **D**. Hypoproct and paraprocts. Scale bar: 1 mm.

**Figure 13.**
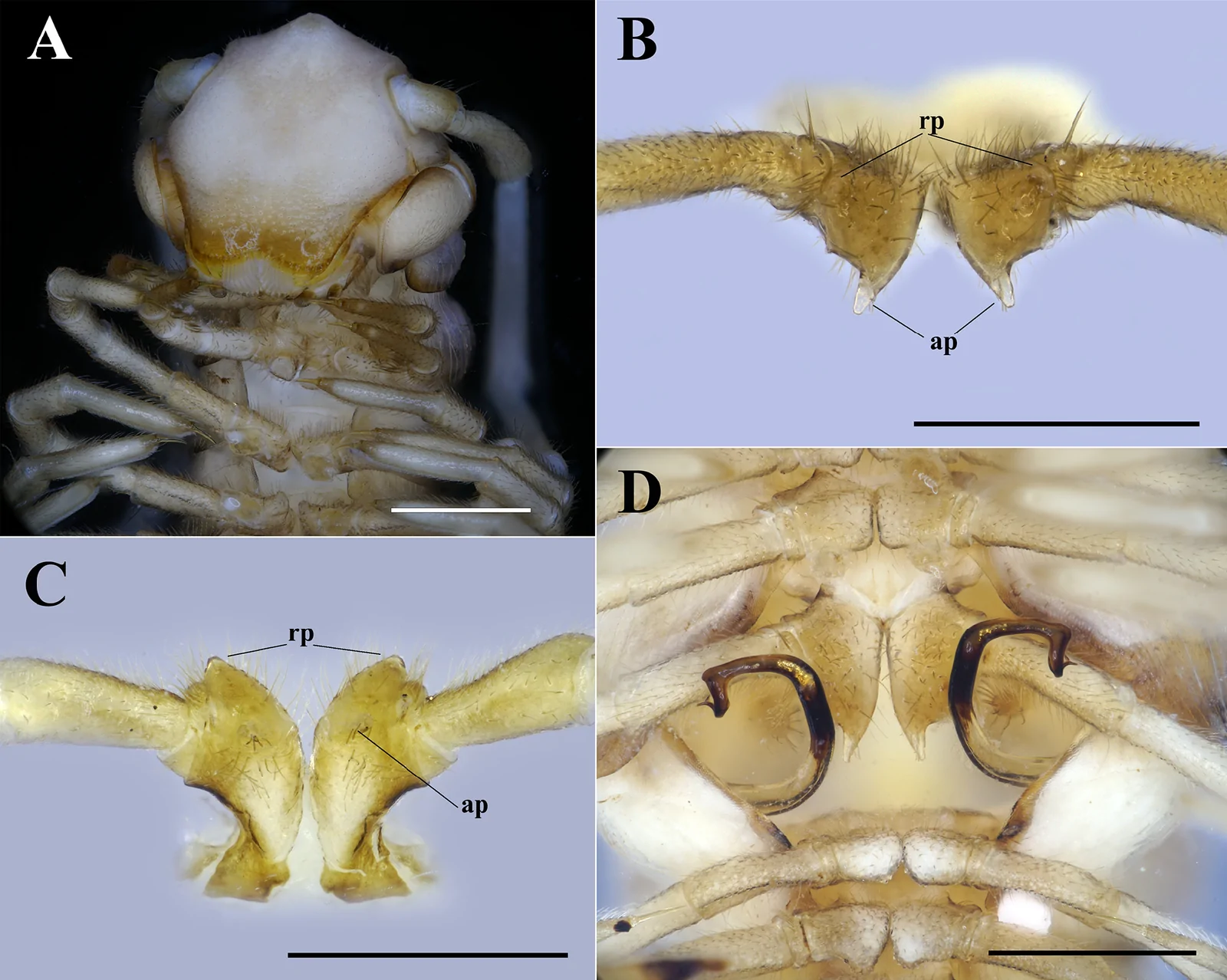
*Vietpetalum simplex* sp. nov., holotype, IEBR-Myr 1070H. **A**. First and second pairs of legs; **B, C**. Legpair 7, coxa, trochanter and part of the prefemur, ventral view (**B**), posterior view (**C**); **D**. Gonopod in gonocoel, ventral view. Scale bar: 1 mm.

**Figure 14.**
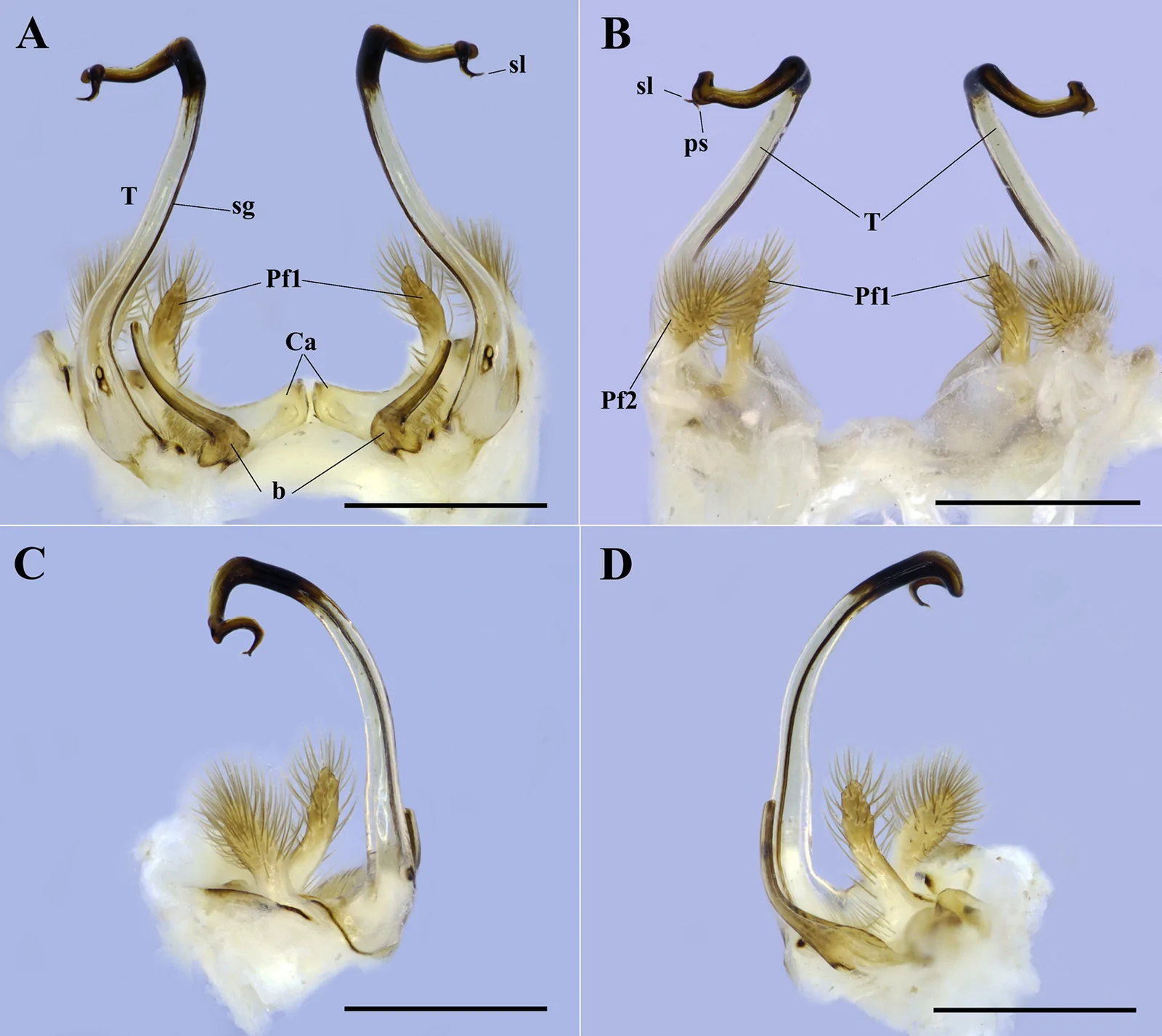
*Vietpetalum simplex* sp. nov., holotype, IEBR-Myr 1070H. **A, B**. Gonopods, ventral view (**A**), dorsal view (**B**); **C, D**. Right gonopod, lateral view (**C**), mesal view (**D**). Scale bar: 1 mm.

**Figure 15.**
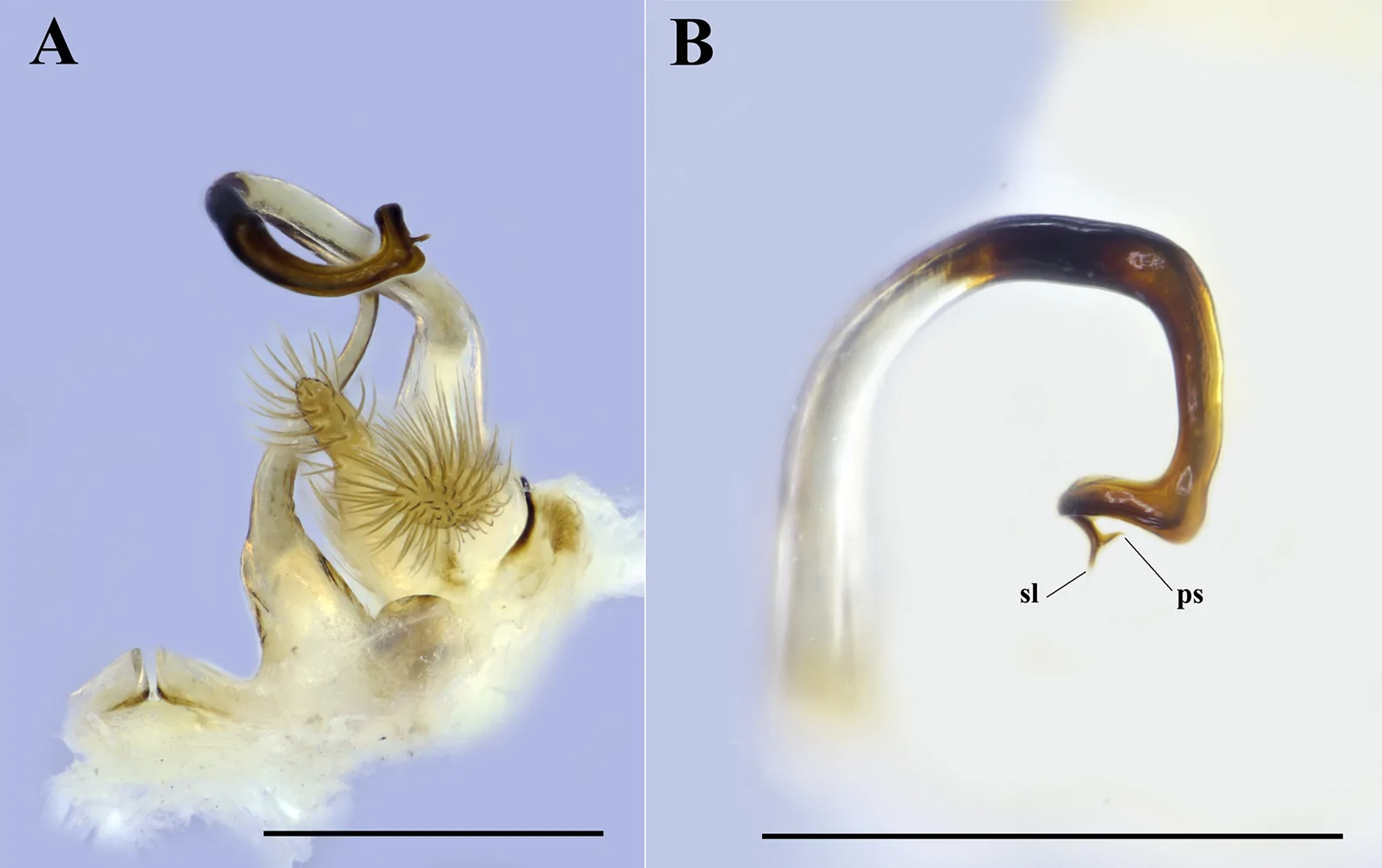
*Vietpetalum simplex* sp. nov., holotype, IEBR-Myr 1070H. **A**. Right gonopod, anterior view; **B**. Tip of right telopodite. Scale bar: 1 mm.

**Figure 16.**
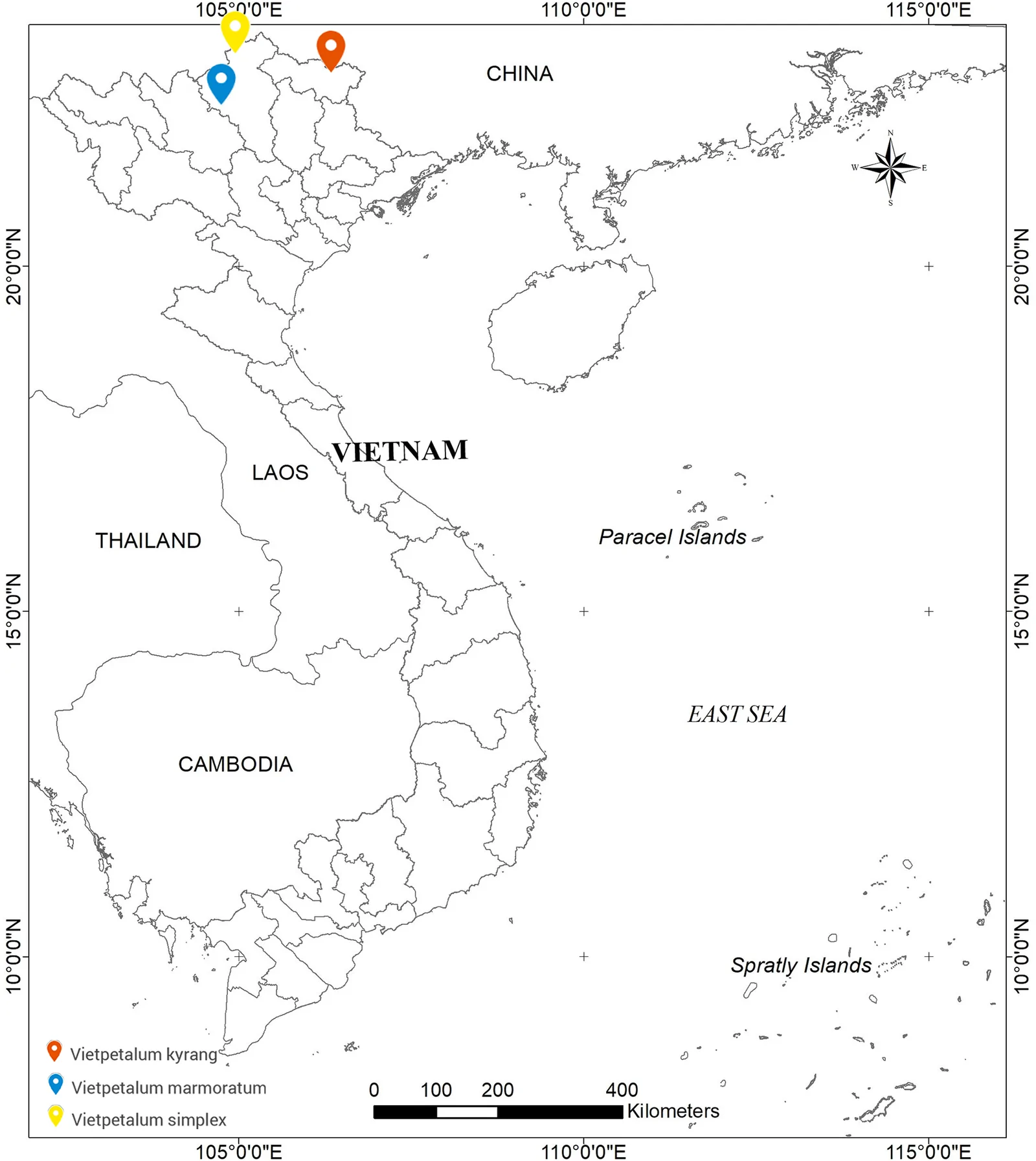
Distribution of the genus *Vietpetalum* gen. nov.

***Paratypes***: Vietnam • 1 male, 1 subadult male, 2 females, 4 juveniles; same data as holotype; IEBR-Myr 1070P.

##### Differential diagnosis.

This species of *Vietpetalum* differs from congeners in having the gonopods *in situ* strongly curved and C-shaped (Fig. 13D), the subfalcate coxal process (b) slenderer and shorter, and the telopodite (T) without a conspicuous distal lamella (dl).

##### Etymology.

The specific epithet is a Latin noun, used in apposition, and emphases the simplicity of gonopods.

##### Description.

**Male holotype**: length about 52.4 mm; width and height of midbody PT 3.0 mm and 2.6 mm, respectively; 59 PT+ telson.

***Colour***: living specimens brownish yellow. Ethanol preserved specimens (Fig. 9D): generally whitish yellow; posterior part of metazonites with a brown posterior margin; dorsal crests blackish brown; head, pleurotergites, antennae, and telson whitish yellow; legs yellowish brown.

***Head*** (Fig. 10A–C) modified; frons with small projection; forehead and vertex concave.

***Antennae*** (Fig. 11B) long, extending beyond posterior edge of PT 7 when folded backwards; ratio antenna/body length about 1/7; all antennomeres white; length of antennomeres: 1 = 0.4 mm; 2 = 1.6 mm; 3 = 1.7 mm; 4 = 1.4 mm; 5 = 1.5 mm; 6 = 1.00 mm; 7 = 0.3 mm; tip of antennomere 7 with four cones protruding well beyond edge (broken in Fig. 11B).

***Field of ommatidia*** (Fig. 11A) black, well delineated, composed of 38 ommatidia in four or five horizontal rows. Tömösváry’s organ about four times larger than largest ommatidium, placed between eye and base of antenna (Figs 10A, 10B, 11A).

Width of PT: 6>>7>8–14>5>4=3=2>1. PT slightly broader than high; height of 10^th^PT: 2.5 mm, width 2.9 mm.

***Collum*** (Fig. 10A, B) much narrower than head; pleurotergite 6 in males strongly enlarged (Fig. 10D). Crests on collum (Fig. 10A, B) moderately expressed mostly in posterior part of ring. Complete crests series appearing from PT2 onwards. Above ozopores, midbody PT with 3+3 primary crests and with 3+3 secondary short crests between primary crests (Fig. 11C, D); 3^rd^ primary crest strongly enlarged, other primary crests not flattened, almost equally broad along metazonal length; secondary crests shorter and slightly posteriorly narrowed. Ozopores lying on primary crest 3, visible from sixth to last but one PT (Fig. 11C, D).

Below ozopores, midbody PT with 2+2 primary crests and 2+2 shorter and thinner secondary crests between primary crests, and 10–11 lower crests down to ventral pleurotergal edge (Fig. 11D).

Midbody pleurotergal setae 5+5, located at caudal edges of primary crests (Fig. 11C, D); setal pattern as in Table 2. Axial line obviously distinct.

**Table 2.** Chaetotaxy in *Paracortina simplex* sp. nov.

	**Anterior setae**	**Posterior setae**
Collum	5+5	—
PT2	5+5	—
PT3	5+5	—
PT4	5+5	—
PT5	2+2	3+3
PT6–penultimate PT		5+5 (6–7+6–7 on several posterior PT)

***Epiproct*** (ep) (Fig. 12B–D) simple, with 7+7 setae on lateral sides and 5+5 setae in transverse rows on dorsum. Hypoproct (hp) (Fig. 12D) tripartite, medial sclerite largest, subtrapeziform, bearing two paramedian macroseta; each lateral sclerite with a single macroseta born on a large tubercle. Paraprocts (pp) (= anal valves) (Fig. 12D) smooth, each divided into a small upper and large lower sclerite, both with a pair of macrosetae. Spinnerets long and slender, tube-shaped, each ending with a long seta. All setae on telson brown, contrasting with yellowish background.

***Male leg-pairs*** 1 and 2 much shorter, with strong setae on ventral side of femorite and tibia, leg-pair 3 slightly shorter than following legs; tarsal pads large clearly visible on pregonopodal legs, then gradually thinner and eventually absent on subsequent legs. All legs ending with a rather slender, long, curved claw. Coxal sacs present from legs 3–5 (PT 9).

Coxa 2 with a pair of small anterior process directed anteriad, a pair of gonopores placed on small cones (Fig. 13A). Coxa 6 normal, without processes or modifications. Coxa 7 (Fig. 13B, C) with anterior round lobe-like process (rp) directed ventrad, and an angular process (ap) directed posteriad.

***Gonopods*** (Figs 13D, 14, 15) yellowish brown, some parts (seminal groove, distal part of telopodite) dark brown to black. Gonopods protruding well beyond the gonocoel, stems of telopodites long, thin, *in situ* C-shaped, subparallel in part, distally diverging and pointing laterad (Fig. 13D). Each gonopod with two small, clavate prefemoroidal processes (Pf1 and Pf2) densely covered apically with long macrosetae (Fig. 14). Coxa with a round lobe (Ca) on mesal side and a slender, short process (b) (Fig. 14), with a blunt truncated (or broken) apex. Telopodite (T) long, slender, *in situ* apically twisted laterad and distal lamella absent (Fig. 15). Distal part of telopodite sinuous, smoothly narrowing to end at solenomere (sl) and parasolenomere (ps) (Fig. 15B). Seminal groove terminating in solenomere (Fig. 15B).

##### Variations.

Other male has 54 PT + telson; body length of 49.6 mm; midbody width of 2.9–3.0 mm and height of 2.4–2.5 mm. The subadult males have 42–58 PT +telson, body length of 42.2–48.9 mm.

##### Females.

No female specimens have been found for the study.

##### DNA barcode.

The COI barcode is uploaded to GenBank with an accession number QB114523. The new species has a genetic similarity of 89.45% with *Paracortina kyrang*.

##### Cave habitat.

Cave is located close to the Vietnam–China border in Tuyen Quang Province, northern Vietnam, at the elevation of 1,800 m a.s.l. The cave entrance is small and narrow, forming a tunnel for the first 20 m before widening inside. Darkness begins approximately 3 m from the entrance. The cave floor is predominantly wet and covered with small rocks; no clay deposits were observed in the area where the species was discovered. The new species was found on the cave wall and on large rocks 20 m from the cave’s entrance. Several other millipede species were also present in the cave, including *Eutrichodesmus* sp., polydesmids, and julids.

##### Distribution.

The species is known only from its type locality.

### Identification key to the genera of the family Paracortinidae based on gonopod structures

**Table:** 

1	Gonopod without subfalcate coxal process b	*** Scotopetalum ***
–	Gonopod with a subfalcate coxal process b	**2**
2	Gonopods parallel, telopodites stout with their distal parts crossing. Mesal coxal process b broad and almost of the same size as telopodite	*** Crassipetalum ***
–	Gonopods parallel, distal part diverging. Mesal coxal process b thinner and much shorter than telopodite	**3**
3	Telopodite with a pointed triangular projection at the third to mid-length of its posterior margin, marking an abrupt twist and narrowing of the process	*** Angulifemur ***
–	Telopodite without a pointed triangular projection at the third to mid-length of its posterior margin	**4**
4	Telopodite sinuous or curved, and *in situ* bent at an angle of 90 degrees of the main branch; distal part strongly narrowed with only a reduced to absent distal lamella	***Vietpetalum* gen. nov**.
–	Telopodite stouter gently curving distad; distal part expanding with obvious serrated lamella and additional processes	*** Paracortina ***

### Identification key to the species of the genus *Vietpetalum* based on gonopod structures

**Table:** 

1	Gonopods *in situ* mostly parallel, distal part diverging (Fig. 5D; Nguyen et al. 2023: fig. 2A), subfalcate coxal process (b) stout and longer reaching the distal part of the telopodite; telopodite (T) strong, with distal lamella (dl)	**2**
–	Gonopods *in situ* strongly curved, C-shaped (Fig. 13D), subfalcate coxal process (b) slenderer and short; telopodite (T) slender, without a consipucous distal lamella (dl) (Figs 14, 15)	***V. simplex***
2	Each gonopod with two clavate prefemoroidal processese (Pf1 and Pf2); telopodite (T) sinusoidal, with a distomesal spine (sp), marking two twists distally, and strongly constricted before apex (Nguyen et al. 2023: figs 6, 7)	***V. kyrang***
–	Each gonopod with a long, subfalcate prefemoroidal process (Pf); telopodite (T) curved with no distomesal spine, distally marking a 90° angle with downturned apex (Figs 6, 7)	***V. marmoratum***

## Discussion

DNA barcoding has become a standard tool in modern taxonomy, providing a genetic reference point that complements morphological descriptions (Hubert and Hanner 2015; Rani et al. 2026). Unfortunately, molecular data for species of *Paracortina*, *Crassipetalum*, and *Angulifemur* remain unavailable, as most paracortinid taxa were described before DNA barcoding became a common practice when describing species. Although recent technologies have made it possible to extract genetic information from museum specimens, this process remains challenging because the DNA in these specimens is often fragmented and degraded. In this context, the COI sequences obtained for three *Vietpetalum* species represent a valuable tool for species delimitation. These sequences also provide important information in support of building a library of DNA barcodes for this taxonomic group, and contribute to the understanding the phylogenetic relationships within the millipede family Paracortinidae.

Vietnam’s limestone cave systems represent one of the most remarkable karst landscapes in Southeast Asia, covering approximately 60,000 km^2^ in four major subregions: Viet Bac region (Tuyen Quang, Cao Bang, Lang Son), Northeastern Vietnam (Quang Ninh), Northwestern Vietnam (Lai Chau, Lao Cai, Son La, Hoa Binh, Ninh Binh), and North-central Vietnam (Quang Tri) (Tuyet 2001). The isolation of limestone outcrops creates ecological “islands,” fostering high levels of endemism among plants, land snails, and cave-adapted invertebrates. Numerous millipede species have been recently described from caves, for example *Eutrichodesmus* species (Golovatch et al. 2009, 2016), *Glyphiulus* species (Golovatch et al. 2007a, 2007b, 2011), callipodidans (Stoev and Geoffroy 2004; Nguyen et al. 2023, 2025), polydesmidans (Nguyen et al. 2024), and paradoxosomatids (Golovatch 2019). The discovery of two additional new species and a new genus further underscores the extraordinary biodiversity concealed within Vietnam’s karst ecosystems. Given the unique characteristics of northern Vietnam’s karst regions, it is highly likely that many more undescribed taxa remain to be found. Intensive surveys will undoubtedly reveal additional species, highlighting the global significance of these cave systems for evolutionary novelty and biodiversity.

## Supplementary Material

XML Treatment for Vietpetalum

XML Treatment for Vietpetalum kyrang

XML Treatment for Vietpetalum marmoratum

XML Treatment for Paracortina simplex

## Additional information

### Artificial Intelligence (AI) use

The authors accept full responsibility for the content of the manuscript, including the disclosure of any use of AI.

No AI tools were used in the preparation of this manuscript.

### Author ORCIDs

H.-A. Le https://orcid.org/0009-0004-2756-2393

D.V. Dang https://orcid.org/0000-0002-0352-8340

C.T. Nguyen https://orcid.org/0009-0006-1498-0101

Y.T. Phan https://orcid.org/0000-0001-9628-7584

N. Akkari https://orcid.org/0000-0001-5019-4833

A.D. Nguyen https://orcid.org/0000-0001-9273-0040

### Data availability

All of the data that support the findings of this study are available in the main text.
